# Examining the concordance of retinal ganglion cell counts generated using measures of structure and function

**DOI:** 10.1111/opo.13041

**Published:** 2022-09-06

**Authors:** Victoria Stapley, Roger S. Anderson, Kathryn Saunders, Pádraig J. Mulholland

**Affiliations:** ^1^ Centre for Optometry & Vision Science, Biomedical Sciences Research Institute Ulster University Coleraine UK; ^2^ National Institute for Health Research (NIHR) Biomedical Research Centre at Moorfields Eye Hospital NHS Foundation Trust and UCL Institute of Ophthalmology London UK

**Keywords:** peripheral grating resolution acuity, retinal ganglion cell count, structure‐function relationship

## Abstract

**Purpose:**

There are several indirect methods used to estimate retinal ganglion cell (RGC) count in an individual eye, but there is limited information as to the agreement between these methods. In this work, RGC receptive field (RGC‐RF) count underlying a spot stimulus (0.43°, Goldmann III) was calculated and compared using three different methods.

**Methods:**

RGC‐RF count was calculated at a retinal eccentricity of 2.32 mm for 44 healthy adult participants (aged 18–58 years, refractive error −9.75 DS to +1.75 DS) using: (i) functional measures of achromatic peripheral grating resolution acuity (PGRA), (ii) structural measures of RGC‐layer thickness (OCT‐model, based on the method outlined by Raza and Hood) and (iii) scaling published histology density data to simulate a global expansion in myopia (Histology‐Balloon).

**Results:**

Whilst average RGC‐RF counts from the OCT‐model (median 105.3, IQR 99.6–111.0) and the Histology‐Balloon model (median 107.5, IQR 97.7–114.6) were similar, PGRA estimates were approximately 65% lower (median 37.7, IQR 33.8–46.0). However, there was poor agreement between all three methods (Bland–Altman 95% limits of agreement; PGRA/OCT: 55.4; PGRA/Histology‐Balloon 59.3; OCT/Histology‐Balloon: 52.4). High intersubject variability in RGC‐RF count was evident using all three methods.

**Conclusions:**

The lower PGRA RGC‐RF counts may be the result of targeting only a specific subset of functional RGCs, as opposed to the coarser approach of the OCT‐model and Histology‐Balloon, which include all RGCs, and also likely displaced amacrine cells. In the absence of a ‘ground truth’, direct measure of RGC‐RF count, it is not possible to determine which method is most accurate, and each has limitations. However, what is clear is the poor agreement found between the methods prevents direct comparison of RGC‐RF counts between studies utilising different methodologies and highlights the need to utilise the same method in longitudinal work.

**Key points**

- Poor agreement was found between methods commonly used to estimate retinal ganglion cell counts indirectly, meaning counts can only be compared reliably (longitudinally or across studies) using the same method.
- Retinal ganglion cell counts estimated from functional measures of peripheral grating resolution acuity, where specific subtypes of cells are probed, are lower than those generated using structural data (e.g., optical coherence tomography).
- There was large intersubject variability in retinal ganglion cell counts amongst healthy adult participants; this is being evident regardless of which method was used to obtain the count.

## INTRODUCTION

Retinal ganglion cells (RGC) are the sole output neurons of the retina and have a vital role in maintaining normal visual function. RGC density can be reduced secondary to the physiological expansion of the globe in myopia^1^, ^2^, ^3^ and pathological processes (e.g., glaucoma),^4^, ^5^ with the function of RGCs also being altered in some conditions.^6^ Precise estimates of in vivo RGC density can therefore facilitate the effective detection and monitoring of pathological conditions such as glaucoma, while also permitting the investigation of structure–function relationships.

Whilst methods are being developed to image RGCs in vivo noninvasively using adaptive optics (AO), in combination with a scanning laser ophthalmoscopy (SLO),^7^ optical coherence tomography (OCT)^8^, ^9^, ^10^ or a multimodal system combining both AO‐SLO and AO‐OCT,^11^ significant challenges remain.^12^ For example, there are still difficulties in differentiating RGC subtypes from each other and from displaced amacrine cells (AC).^12^ In addition, studies using such technologies have only been conducted so far on very small samples using specialist equipment not widely available to researchers or clinicians. Therefore, alternative, indirect methods of obtaining person‐specific estimates of RGC numbers have been employed in the literature. These methods utilise average histological measures of RGC density from healthy observers (with or without adjustment for individual ocular biometry), structural measures (e.g., OCT‐derived measures of neural tissues^13^, ^14^) or functional thresholds (e.g., from perimetry^15^, ^16^ or peripheral grating resolution acuity (PGRA) measurements^17^).

Most of our understanding of RGC density and distribution within the retina comes from postmortem histological studies. Curcio and Allen^18^ quantified the RGC cell body (RGC‐CB) density for six human retinas from five young adult donors (aged 27–37 years) without eye disease. Other histological counts have been provided by Sjöstrand et al.,^19^ who reported RGC density along the vertical (90°) meridian for three subjects (aged 39–73) with no history of eye disease, Dacey,^20^ who considered only the midget subtype of RGCs from donor eyes (*n* = 46, ages 16–82 years) and Tribble et al.^21^ for a small sample of older subjects with (*n* = 4, mean age 74.5 years) and without (*n* = 6, mean age 81.6 years) glaucoma. Within the central retina, lateral displacement of RGC‐CBs from their underlying input photoreceptor(s) means there is a discrepancy between the RGC‐CB position and RGC receptive field (RGC‐RF) position.^22^ The RGC‐RF, rather than the cell body, is what is functionally responsible for moderating the localised retinal response to visual stimuli and so should be used in evaluating retinal structure–function relationships. Both Sjöstrand et al.^19^ and Dacey^20^ attempted to measure this lateral RGC displacement, using these measures to derive ‘effective’ RGC densities (i.e., RGC‐RF density) from their raw RGC‐CB data. Curcio and Allen's RGC‐CB data^18^ was used in the development of two theoretical equations that model RGC‐RF density in the human visual field.^22^, ^23^ These models of Drasdo et al.^22^ and Watson^23^ have been used subsequently to determine the number of RGC‐RFs underlying stimuli at select retinal locations.^24^, ^25^

Whilst average histological models have been widely used to estimate RGC numbers in individual observers, their use is markedly limited by the relative inability to account for interindividual variations in RGC density secondary to physiological and/or pathological processes. Other techniques utilising measures of function or structure attempt to overcome such limitations. One ‘function’ method of estimating RGC‐RF count indirectly is by measuring high‐contrast PGRA. Unlike in the fovea, where visual resolution is optically limited, resolution in the peripheral retina is neurally limited.^17^ Specifically, peripheral resolution acuity is determined by RGC spacing, with the result that RGC density can be inferred from psychophysical measurements of PGRA.^1^, ^17^, ^26^, ^27^ The methodology used to convert measurements of PGRA to estimates of RGC‐RF density was outlined by Thibos et al.,^17^ and has since been utilised in more recent clinical studies.^28^, ^29^ This assumes the array of RGCs to be hexagonal^30^, ^31^ and is based on the Nyquist‐Shannon sampling theorem, which states there must be at least two sample points (visual receptors, RGCs) per cycle of a grating in order for it to be resolved correctly.^32^, ^33^

There have also been several mathematical models developed that relate functional measures of perimetric sensitivity to underlying RGC‐RF density or number.^16^, ^34^, ^35^, ^36^, ^37^ The majority of these were developed using the human histological data of Curcio and Allen^18^ as a basis,^16^, ^34^, ^35^ but Harwerth et al.^36^, ^37^ used behavioural and postmortem histological data from adult rhesus monkeys. Their final equations incorporated adjustments for the larger axial length of the human compared with the monkey eye and for the different perimetric testing strategies used for the monkey and human subjects.^36^ These models relating perimetric sensitivity to RGC‐RF number are not included within the present study, as comparisons between these models have been made previously^38^, ^39^ and the present study was conducted on healthy participants with normal visual fields.

Retinal ganglion cell count can also be estimated from structural measurements. For example, Harwerth et al.,^13^ using normative histological counts of RGC axons and OCT‐derived peripapillary RNFL thickness measurements, produced RGC counts that corresponded well with those estimated from behavioural perimetry data in primates. This method is, however, limited by assumptions regarding the topographic relationship between RGC axons at the optic nerve head and the location of the corresponding RGC bodies within the retina.^40^, ^41^ To account for such issues, Raza and Hood^14^ later developed a method to obtain localised RGC counts from OCT‐measured RGC‐layer thickness in the central retina and normative histology RGC density data.^18^ Their method has since been used by other studies to investigate the pattern of normal age‐related loss of RGCs,^42^ and to calculate the number of RGC‐RFs underlying a stimulus for the investigation of structure–function relationships.^43^, ^44^, ^45^, ^46^

In summary, in the absence of a noninvasive direct method of obtaining RGC‐RF counts in an individual, there have been several different methods developed and used within the literature to indirectly estimate RGC counts in a given eye. Despite this, there is limited information as to the agreement between methods. The purpose of this study was to compare the RGC‐RF count underlying an achromatic spot stimulus (Goldmann III [GIII], 0.43°) when estimated in healthy adults using: (i) functional measures (PGRA), (ii) structural measures (RGC‐layer thickness)^14^ and (iii) average histology data modelled to account for interindividual variations in axial length.

## METHODS

### Participants

Forty‐four participants were recruited (age range 18–58 years). The cohort had a wide range of refractive errors (spherical equivalent refraction [SER]) ranging from −9.75 DS to +1.75 DS (mean −1.93 DS), with 24 participants classified as myopic (≤−0.50 DS).^47^ Refractive error and axial length were measured objectively following the instillation of tropicamide hydrochloride 1.0% using a binocular open‐field autorefractor (NVision‐K 5001, Shin‐Nippon, shin‐nippon.jp) and IOLMaster (Carl Zeiss Meditec, zeiss.com), respectively. Axial length ranged from 22.59 to 28.86 mm (mean 24.65 mm). All participants had best corrected (unaided or with their habitual spectacle correction) monocular, distance visual acuity of 0.00 logMAR (6/6 Snellen) or better in each eye. All participants had no ocular abnormalities other than refractive error; intraocular pressure was between 10 and 21 mmHg (Goldman Applanation Tonometry [GAT]); visual fields were full with the 24‐2 SITA standard threshold test (Humphrey Visual Field Analyser, Carl Zeiss Meditec, zeiss.com); and no media opacity or disease was detected with slit lamp examination and fundoscopy. Peripapillary RNFL and macular OCT scans captured with a Spectralis OCT (Heidelberg Engineering GmbH., heidelberg.com) were within normal limits. No participant had a systemic condition or was taking medication known to affect ocular/visual function.

Ethical approval to carry out the data collection was granted from the Ulster University Biomedical Sciences Research Ethics Filter Committee and the research adhered to the tenets of the Declaration of Helsinki. Prior to data collection, informed written consent was obtained from each participant.

### Determining RGC‐RF count

Following the screening process, one eye was randomly selected for experimental measures. RGC‐RF count underlying a standard GIII stimulus (diameter 0.43°, area 0.15 deg^2^) was calculated using: (i) PGRA thresholds, (ii) average histology data for healthy observers adjusted for participant axial length (Histology‐Balloon model) and (iii) the method of Raza and Hood (OCT‐model).^14^ Where required, an eccentricity‐specific conversion factor (q_p_) was used to translate degrees in visual space into millimetres on the retina, so as to calculate both stimulus position (retinal eccentricity in mm) and area (mm^2^). This conversion factor was calculated using the abbreviated axial length method described by Bennett et al.^48^ Briefly, this requires a conversion factor (q_o_) to be calculated for the fovea (q_o_ = 0.01306 * [axial length – 1.82]), with an adjustment then made for the eccentricity (U, in degrees) at which the RGC measurements were estimated (q_p_ = q_o_ – 0.000014 U^2^). For this study, ‘U’ was equal to 8.1 degrees, and the axial length value used (23.84 mm) corresponded to the average axial length of the histology samples from Curcio and Allen.^18^ The consistent use of an emmetropic axial length was appropriate given that PGRA measures were undertaken under conditions of Knapp's Law (see section headed Peripheral Grating Resolution Acuity (PGRA) below), and raw OCT data were adjusted using an observer‐specific conversion to account for ocular magnification effects (see section headed OCT model below).

#### Peripheral grating resolution acuity (PGRA)

Peripheral grating resolution acuity was measured on a gamma‐corrected CRT monitor (SONY 420GS, Sony Corporation, sony.net; pixel resolution, 1280 × 1024, refresh rate 75 Hz, achromatic background 30 cd/m^2^), with a viewing distance of 620 mm. Prior to measurement, a minimum warm‐up period of 1.5 h was allowed. Refractive correction was achieved by placing a full aperture trial lens at the approximate anterior focal point of the eye (15.2 mm,^49^) to ensure retinal image size, in mm, was constant across participants (employing Knapp's Law^50^). The eye not being tested was occluded with an opaque eye patch. Participants were instructed to remain fixated on a central cross stimulus while peripheral stimuli were presented, with fixation monitored manually by the researcher.

Stimuli were generated using MATLAB (2016b, The MathWorks Inc., mathworks.com) with Psychtoolbox (v3.0) and a Bits# (Cambridge Research Systems, crsltd.com). PGRA was measured using achromatic, oblique (45° and 135°) Gabor patches in the sine phase (SD × spatial frequency = 4; Michelson contrast, 99%). The gratings had the same mean luminance as the background (30 cd/m^2^) and were presented for a duration of 500 ms. The stimuli were all presented at 8.1° eccentricity along four primary meridians (90°, 180°, 270° and 360°). This equates to a retinal eccentricity of 2.32 mm for an emmetropic eye having an axial length of 23.84 mm. As Knapp's Law was invoked, the stimuli were presented at the same retinal eccentricity (2.32 mm) for all participants. Responses to indicate the orientation of the grating were collected using a Cedrus RB‐540 response pad (Cedrus Corporation, cedrus.com). If the participant was unable to resolve the grating, they were asked to guess, and only after a response was registered was the next stimulus presented. A 3‐up‐1‐down staircase procedure was used, appropriate for the two‐alternative forced choice paradigm. Spatial frequency was initially set at 5 cycles/degree (c/deg); this being altered by 20% with participant responses when reversals <2, by 10% when reversals = 2 and by 5% when reversals >2. To optimise participant response and minimise any effects of changing spatial summation, the standard deviation of the gaussian window was varied with spatial frequency to maintain four effective cycles (at 99% contrast) within the stimulus, in which the data of Anderson et al.^51^ indicated to be the point at which peripheral resolution acuity plateaus with the number of cycles. The staircase terminated after four reversals, with the threshold calculated as the average of these four reversals. All four locations were tested in a randomly interleaved fashion within a single test run.

The localised threshold spatial frequency values in c/deg were then converted into a minimum angle of resolution (MAR) and transformed into metric units (mm) using the conversion factor q_p_. A constant value of q_p_ was used for all participants, given that Knapp's Law was satisfied during measurements. RGC‐RF density (/mm^2^) was then calculated using equation 1 and multiplied by the area of the GIII stimulus (in mm^2^) to give the number of RGC‐RFs underlying the GIII stimulus for each participant.

(1) $$\mathrm{RGC}\mathrm{‐RF}\;\text{density}={\left(\frac{0.93}{\mathrm{MAR}}\right)}^{2}$$

Equation 1 Calculating RGC‐RF density from PGRA measures.

#### Histology‐balloon model

With this method, the RGC‐RF number for each participant was calculated using the normative, histological RGC counts for an age‐similar cohort^18^ and scaling to simulate a simple global expansion (‘balloon’) model of myopia. A global expansion model of myopia assumes that the total number of RGCs remains constant, but that local RGC density is uniformly and proportionally reduced secondary to axial elongation and retinal stretch.

**FIGURE 1:**
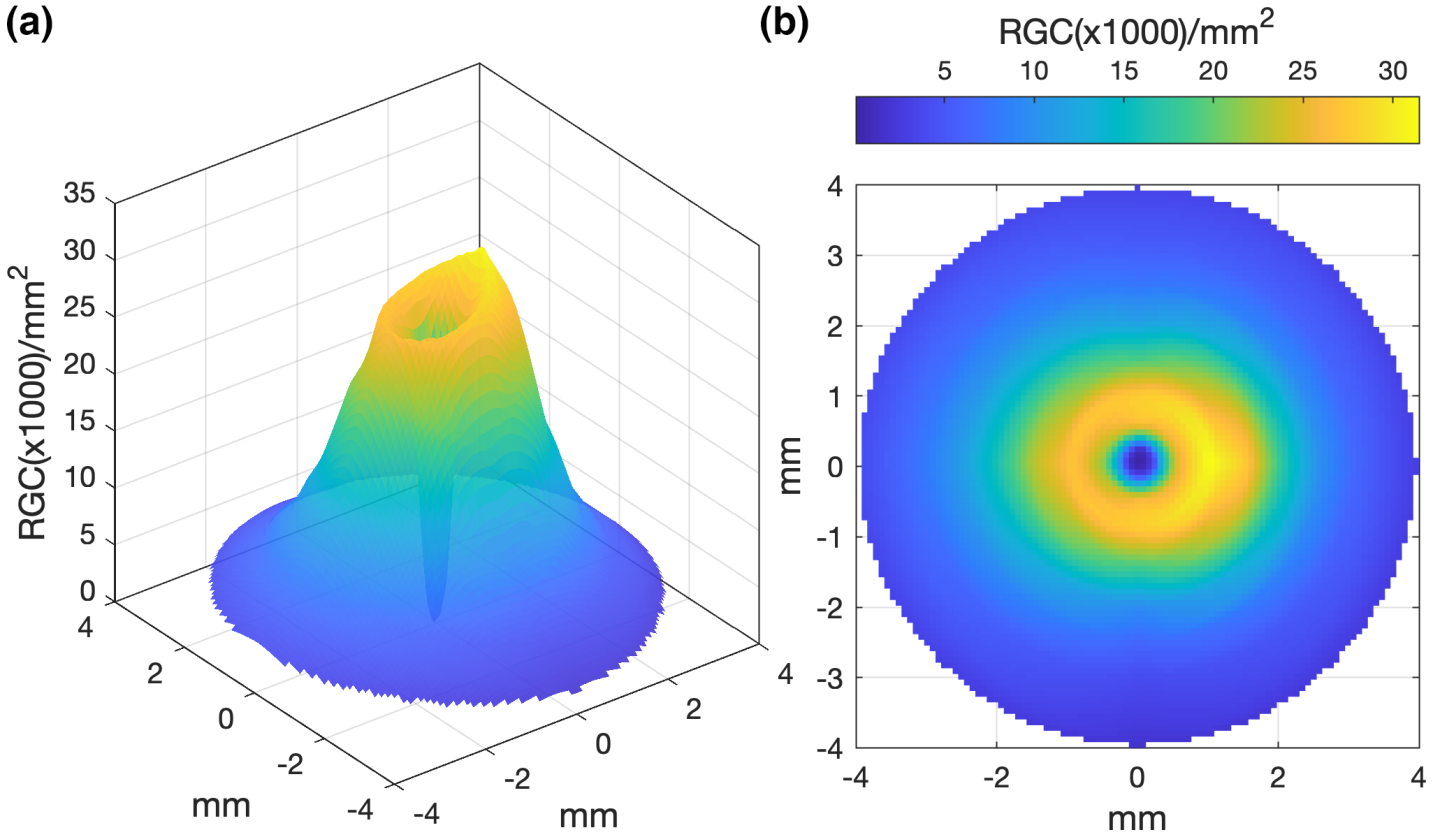
Interpolated histological retinal ganglion cell (RGC) density values (per mm^2^) of Curcio and Allen^18^ presented in (a) three‐dimensional, and (b) two‐dimensional forms for the right eye.

First, Curcio and Allen's histology RGC counts (RGC/mm^2^) for the four primary meridians were linearly interpolated along polar coordinates to generate estimates of RGC/mm^2^ at 10,000 locations across the central retina (Figure 1), similar to the approach of both Garway–Heath et al.^34^ and Raza and Hood.^14^

**FIGURE 2:**
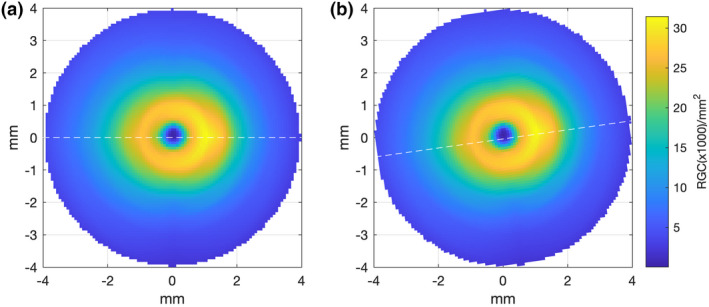
(a) Retinal ganglion cell (RGC) en‐face density plot with both the fovea and optic nerve falling along the horizontal meridian (dashed white line), reflecting the orientation of the original histology data of Curcio and Allen.^18^ (b) Example of an individual's density plot where the orientation has been adjusted to reflect their measured angular subtense between the fovea and optic nerve (in this case 8^º^, dashed white line)

**FIGURE 3:**
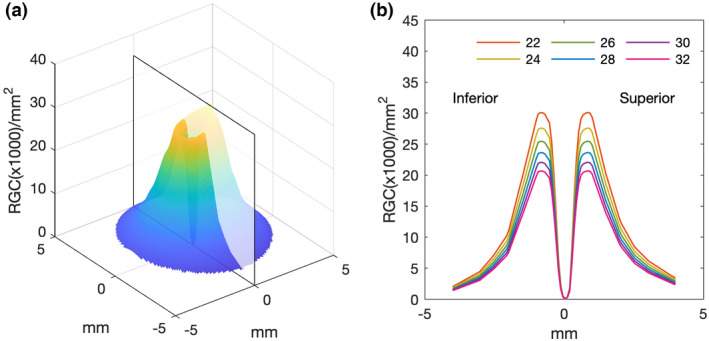
(a) Retinal ganglion cell (RGC) density plot for locations within the central retina, using extrapolated data from Curcio and Allen's RGC counts.^18^ (b) Plot demonstrating simulated variations in RGC density along the vertical meridian (translucent plane in (a)) for changes in axial length (22 to 32 mm). Simulated RGC counts are calculated assuming an emmetropic axial length of 23.84 mm and a uniform expansion of the globe with a constant number of RGCs.

As the histological data of Curcio and Allen^18^ are presented with the fovea and optic nerve head (ONH) both lying along the horizontal meridian (Figure 2a), the interpolated RGC/mm^2^ map was rotated to reflect the true anatomical location of the ONH relative to the fovea for each participant, according to the OCT‐measured angular subtense between their macula and ONH centre (Figure 2b). The histological RGC/mm^2^ values were then proportionally scaled according to the degree to which each observer's axial length varied from the mean axial length of the histology samples,^18^, ^52^ assuming a global expansion model of myopia [scaling factor = 23.84/ participant's axial length] (Figure 3).

The number of RGC‐RFs underlying the GIII stimulus presented at 8.1° in the visual field (same location as the PGRA method) was subsequently calculated as the product of the mean histologically derived RGC/mm^2^ values over the area of the stimulus in mm^2^. The retinal position (in mm) and size (in mm^2^) of the stimulus were determined using the conversion factor q_p_ and adjusted for the lateral RGC displacement from underlying photoreceptors. The latter was calculated using the method proposed by Drasdo et al.^22^ incorporating the recent adjustments proposed by Montesano et al.,^46^ including customised displacement values based on an individual's axial length and independently displacing every point along the edge of the stimulus.

#### OCT‐model

The method used to obtain RGC‐RF count from OCT‐derived RGCL‐thickness measurements was based on the method outlined by Raza and Hood.^14^ The first steps of this method are identical to those described above for the Histology‐Balloon method; Curcio and Allen^18^ histological RGC density data (RGC/mm^2^) was interpolated (Figure 1), rotated (Figure 2) and scaled (Figure 3) assuming a global expansion model of myopia. The next step of the OCT method requires ganglio cell layer‐ (GCL‐) layer thickness measurements, obtained by taking a 30° × 25° posterior pole scan centred on the fovea with the Spectralis OCT. The RGC‐layer was analysed over a 24° × 24° grid area, segmented using inbuilt software, with any errors in this automated segmentation corrected manually. OCT data were exported as RAW files (.vol) using the Heidelberg Eye Explorer (Heidelberg Engineering, business‐lounge.heidelbergengineering.com) and then imported into MATLAB where custom‐written code was used to determine the RGC‐RF count. The transverse scaling of the OCT data was adjusted using an observer‐specific conversion factor, q, to account for ocular magnification effects.

The interpolated, rotated, scaled histology RGC density map (RGC/mm^2^) was then converted into volumetric density (RGC/mm^3^) by dividing the data point‐by‐point by the average RGCL thickness (mm) measured for the non‐myopic (control) participants. In line with the original method,^14^ a ‘leave‐one‐out’ approach was implemented when estimating the RGC number for a control participant, whereby the average RGCL thickness was calculated using all other control participants. Stimulus size and position on the retina were calculated in the same way as for the Histology‐Balloon method. To calculate an individual's RGC‐RF number underlying a stimulus, the volumetric density data [RGCD, RGC/mm^3^] was then convolved with the individual's co‐localised RGC‐layer thickness [RGCL, mm] and the stimulus area [S‐area, mm^2^] using equation 2:

(2) RGC‐RF=RGCL·RGCD·S‐area

Equation 2 OCT‐model RGC number calculation.

### Comparisons with published unscaled‐histology models and theoretical equations

All histology data were taken from the original paper (unless stated otherwise, see Table 1) using GraphClick software (version 3.0.3, Arizona Software Inc., arizona‐software.ch/).^18^, ^19^, ^21^, ^27^ Data were taken at a retinal eccentricity of 2.32 mm or the equivalent angular eccentricity (note that in the absence of axial lengths stated for the histology samples, the q_p_ value based on an axial length of 23.84 mm was used to convert between mm and degrees).

**TABLE 1:** Obtaining RGC counts from histology papers for comparison. The data for Dacey^20^ were taken from Anderson et al.^27^

Label	Data from	RGC Type	RGC‐CB or RF	RGC location
Curcio	Fig. 6, Curcio and Allen^18^	All	CB	4 primary meridians
Sjöstrand	Fig. 5, Sjöstrand et al.^19^	All	RF	Average of superior and inferior meridians
Dacey	Fig. 3, Anderson et al.^27^	Midget	RF	Temporal retina
Tribble	Fig. 1C (left panel; healthy), Tribble et al.^21^	All	CB	Average

Abbreviations: CB, cell body; RF, receptive field; RGC, Retinal ganglion cell.

Comparisons were also made to values of the RGC‐RF number, both total and midget‐only populations, obtained using the theoretical equations of Drasdo et al.^22^ and Watson.^23^ For the Drasdo et al.^22^ model, their appendix equations 6 and 7 were used to obtain an estimate of RGC‐RF density (cells/deg^2^) for each primary meridian separately. For the Watson^23^ model, estimates of RGC‐RF density (cells/deg^2^) were obtained for the four primary meridians of the right eye visual field using their interactive Retinal Topography Calculator. This calculator also allows one to specify the density for the ON‐ or OFF‐midget cells separately. These density values are included and referred to as the density of ‘ON’ midget cells in our results, but the values could also apply to the ‘OFF’‐midget cells for the retinal locations examined. For both models, RGC‐RF density was given as cells/deg^2^ and so these values were converted into RGC‐RF/mm^2^ using appendix equation 7 from Watson's paper.^23^

### Statistical analysis

Statistical analysis was carried out using MATLAB (2020, The MathWorks Inc, mathworks.com) and R‐Studio (Version 3.6.2, rstudio.com). The normality of each data set was assessed using a Shapiro–Wilk test, with the appropriate nonparametric tests applied where indicated. For all statistical tests, an alpha value of 0.05 was considered statistically significant, with Holm–Bonferroni correction applied as necessary.

## RESULTS

### Comparison of the RGC‐RF count estimates

**FIGURE 4:**
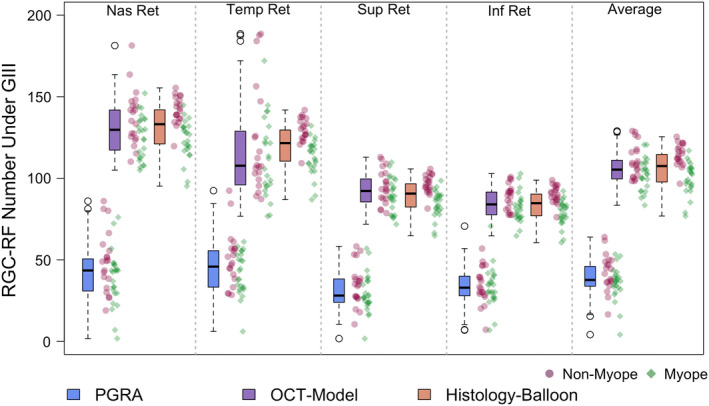
Retinal ganglion cell receptive field (RGC‐RF) number underlying a Goldmann III stimulus for the same cohort determined using three different methods at four peripheral retinal locations (all at 2.32 mm retinal eccentricity). Individual data points are included for reference for myopic (green diamonds) and non‐myopic (pink spots) participants. Boxplots follow standard convention: bold line = median, edges of box = interquartile range (IQR). Maximum whisker length is q1‐1.5*(q3‐q1) to q3 + 1.5*(q3‐q1), where q1 and q3 are the 25th and 75th percentiles, respectively. If all values are within these limits, upper and lower whiskers are maximum and minimum values in data set. All boxplots in this paper follow the same conventions. Abbreviations: OCT, optical coherence tomography; PGRA, Peripheral grating resolution acuity.

RGC‐RF count was determined separately at each of the four test locations for the PGRA method, OCT‐model and Histology‐Balloon model, with an average of the four locations also being calculated for each method. The descriptive statistics (median, IQR) for each location are included in Table 2, with results displayed graphically as boxplots in Figure 4. The individual data points are also shown; stratified according to refractive group (i.e., ‘myopes’ SER ≤−0.50 DS and ‘non‐myopes’ SER ≥−0.25 DS).

**TABLE 2:** RGC‐RF number underlying a GIII stimulus determined using three different methods

Retinal Location (2.32 mm eccentricity)	PGRA	OCT‐Model	Histology‐Balloon
Nasal	43.5 [30.9–50.6]	129.7 [117.3–141.9]	133.2 [121.1–142.0]
Temporal	45.9 [33.3–55.6]	107.7 [95.9–128.9]	121.6 [110.5–129.6]
Superior	28.1 [24.0–38.3]	92.2 [85.5–99.6]	90.6 [82.4–96.6]
Inferior	32.9 [28.0–40.0]	84.0 [77.7–91.5]	84.7 [77.0–90.3]
Average across all meridians	37.7 [33.8–46.0]	105.3 [99.6–111.0]	107.5 [97.7–114.6]

*Note*: Results displayed as median [IQR].

Abbreviations: OCT, optical coherence tomography; PGRA, Peripheral grating resolution acuity.

At all locations the RGC‐RF number appeared to be dependent upon the method used to obtain the estimate (Figure 4, Friedman test: nasal χ^2^(2) = 66, *p* < 0.001; temporal χ^2^(2) = 68.2, *p* < 0.001; superior χ^2^(2) = 66.7, *p* < 0.001; inferior χ^2^(2) = 66.2, *p* < 0.001; average of all lo χ^2^(2) = 66, *p* < 0.001). Whilst the median RGC‐RF values appear similar for the OCT‐model and the Histology‐Balloon model (average across all locations: OCT‐model median 105.3, IQR 99.6–111.0; Histology‐Balloon model median 107.5, IQR 97.7–114.6), RGC values obtained using the PGRA method (average across all locations median 37.7, IQR 33.8–46.0) were markedly lower than both the OCT‐model (64% lower) and Histology‐Balloon model (65% lower). For all locations, *post‐hoc* Wilcoxon signed‐rank tests were significant at *p* < 0.001 for comparisons involving PGRA, but nonsignificant (*p* > 0.05) for comparisons between the OCT‐model and Histology‐Balloon model.

Consistent with published histology^18^ and psychophysical literature,^53^, ^54^ for all three methods the RGC‐RF count was significantly greater (all *p* < 0.001, paired *t*‐test) when averaged across the two horizontal retinal locations (mean ± SD; PGRA 44.5 ± 14.4; OCT‐model 123.7 ± 17.2; Histology‐Balloon 126.0 ± 13.4) compared with the count averaged across the two vertical retinal locations (PGRA 32.9 ± 10.4; OCT‐model 88.5 ± 8.3; Histology‐Balloon 86.7 ± 9.3). On an individual basis, this trend was evident in 91% of participants for the PGRA method, and 100% for both the OCT‐model and Histology‐Balloon methods.

The degree to which the RGC‐RF counts varied across the four meridians (intraretinal variability) was dependent upon the method used. For each participant, intraretinal variability was calculated as the ratio of their maximum to minimum RGC‐RF count across the four test locations. Given that the Histology‐balloon model used the same histological RGC density map for all participants, and then applied the same axial length‐based expansion to each meridian, all participants had the same 1.6‐fold range in RGC‐RF across the visual field. On average, the OCT‐model results showed the same intraretinal range as the Histology‐balloon (1.6‐fold range [IQR 1.5–1.8]) but with the ratio ranging from 1.2 to 2.4 within the cohort studied. RGC‐RF counts from the PGRA method showed the highest degree of intraretinal variability, with a median 1.9‐fold range. In addition, the degree of intraretinal variability also showed the most intersubject variability for PGRA; whilst the lower 25th quartile (1.5‐fold) and minimum (1.2‐fold) were the same as for the OCT‐model, the upper 75th quartile (2.4‐fold vs. 1.8‐fold for OCT) and maximum (4.1‐fold vs. 2.4‐fold for OCT) were higher.

Retinal ganglion cell density (cells/mm^2^) is known to be influenced by myopia,^1^, ^2^, ^3^ with a significant correlation between RGC‐RF number and refractive error observed for all three methods in this cohort (Spearman's Rank Correlation; PGRA ρ = 0.36, *p* = 0.02; OCT‐model ρ = 0.35, *p* = 0.02; Histology‐balloon model ρ = 0.80, *p* < 0.001). To determine whether the inclusion of myopic participants was influencing the overall trends observed, we repeated the analysis for non‐myopic and myopic participants separately for data averaged over the four test locations. Similar trends were observed when considering the myopic and non‐myopic groups separately, albeit with lower RGC‐RF values in the myopic group (see Table 3 for medians and IQR for each method). For both groups, statistically significant intermethod differences in RGC‐RF counts were observed (Friedman test, myopes χ^2^(2) = 36.8, *p* < 0.001; nonmyopes χ^2^(2) = 30.9, *p* < 0.001). As with the whole cohort analysis, median RGC‐RF counts were similar for the OCT‐model and Histology‐Balloon (*post‐hoc* Wilcoxon signed‐rank test: myopes Z = 1.34, *p* = 0.36; nonmyopes Z = −1.34, *p* = 0.36), and PGRA RGC‐RF counts were lower than both the OCT‐model (myopes by 64%; nonmyopes by 60%) and Histology‐Balloon (myopes by 63%; nonmyopes by 62%), with all *post‐hoc* Wilcoxon signed‐rank tests involving PGRA revealing statistically significant differences (myopes OCT/PGRA Z = 4.29, *p* < 0.001, Histology‐Balloon/PGRA Z = 4.29, *p* < 0.001; non‐myopes OCT/PGRA Z = 3.92, *p* < 0.001, Histology‐Balloon/PGRA Z = 3.92, *p* < 0.001).

**TABLE 3:** RGC‐RF number underlying a Goldmann III stimulus determined for all participants and the nonmyopic and myopic groups separately

	PGRA	OCT‐model	Histology
All	Median: 37.7	Median: 105.3	Median: 107.5
	IQR: 33.8–46.0	IQR: 99.6–111.0	IQR: 97.7–114.6
	Range: 4.1–64.0	Range: 83.5–129.0	Range: 76.9–125.5
	Ratio: 15.4	Ratio: 1.5	Ratio: 1.6
Non‐myopes	Median: 43.0	Median: 108.7	Median: 114.3
	IQR: 36.3–49.3	IQR: 101.5–117.2	IQR: 110.3–120.1
	Range: 16.6–64.0	Range: 98.3–129.0	Range: 96.7–125.5
	Ratio: 3.9	Ratio: 1.3	Ratio: 1.30
Myopes	Median: 36.8	Median: 102.6	Median: 100.8
	IQR: 32.4–40.1	IQR: 93.7–109.1	IQR: 94.8–106.2
	Range: 4.1–53.5	Range: 83.5–120.6	Range: 76.9–116.9
	Ratio: 12.9	Ratio: 1.4	Ratio: 1.5

*Note*: Results are displayed for data averaged across all test locations and include median, interquartile range (IQR) and full range. The ratio is calculated as the maximum value/ minimum value and gives an idea of intersubject variability.

Abbreviations: OCT, optical coherence tomography; PGRA, Peripheral grating resolution acuity.

Table 3 also quantifies the inter‐subject range of RGC‐RF counts within the cohort. Considering all participants, there was a 15.4‐fold range in RGC‐RF counts using PGRA as a basis for generating estimates and an ~1.5‐fold range for the other two methods. For all three methods, the intersubject variability was greater for the myopic participants (PGRA 12.9‐fold range, OCT‐model 1.4‐fold range, Histology‐Balloon 1.5‐fold range) compared with the non‐myopic participants (PGRA 3.9‐fold range, OCT‐model 1.3‐fold range, Histology‐Balloon 1.3‐fold range). The largest difference between myopic and non‐myopic participants appeared using the PGRA method.

**FIGURE 5:**
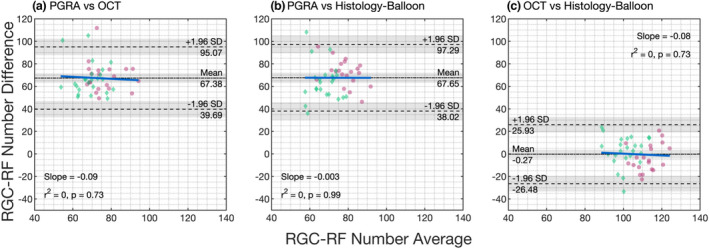
Bland–Altman plots for each pairwise comparison of the three methods used to determine retinal ganglion cell receptive field (RGC‐RF) counts in this study. The mean (black dotted line) and upper and lower limits of agreement (black dashed lines) are shown on the plots. The grey‐shaded area represents the 95% confidence intervals of the mean bias measure and limits of agreement. For reference, data from myopic individuals are shown as green diamonds and for non‐myopes as pink circles. The proportional bias slope is plotted as a solid blue line. Abbreviations: OCT, optical coherence tomography; PGRA, Peripheral grating resolution acuity.

Agreement between the three methods was assessed using Bland–Altman analysis for data averaged across all four meridians (Figure 5). There was a large mean difference in the RGC‐RF count between the PGRA and OCT‐model methods (mean difference 67.4 RGC‐RFs) and between the PGRA and Histology‐Balloon methods (mean difference 67.7 RGC‐RFs). A small difference was found for the Histology‐Balloon and OCT‐model methods (mean difference 0.27 RGC‐RFs). However, despite this, there were wide and similar 95% limits of agreement (LOA) for each pairwise comparison; PGRA/OCT: 55.4; PGRA/Histology‐Balloon: 59.3; OCT/Histology‐Balloon: 52.4.

### Comparisons with histology and theoretical equations

**FIGURE 6:**
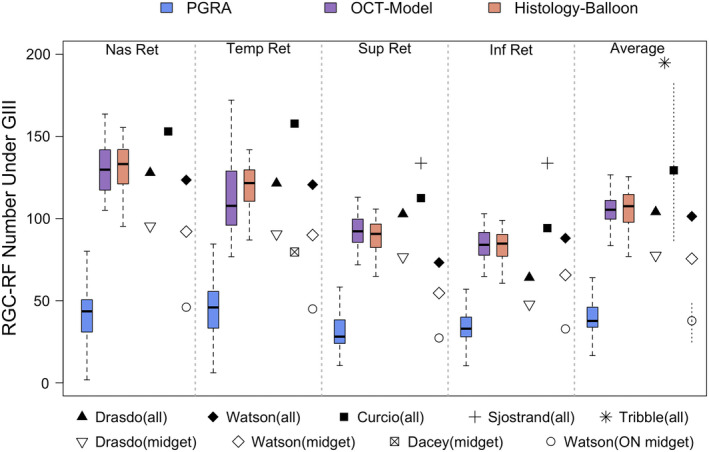
Retinal ganglion cell receptive field (RGC‐RF) number underlying a Goldmann III stimulus calculated in our study using three methods, in comparison with previous histology estimates and values obtained from theoretical equations. NB: Watson's ON‐midget subtype values^23^ are also representative of the OFF‐midget subtype for the retinal location examined. The range of Curcio and Allen's histology data^18^ (averaged across all meridians at the test retinal eccentricity) is shown as dotted vertical lines. The same proportional range has also been applied to Watson's ON‐midget estimate^23^ (which is based on Curcio and Allen's mean total RGC count^18^), to give the theoretical range for ON‐RGCs too. Abbreviations: OCT, optical coherence tomography; PGRA, Peripheral grating resolution acuity. The studies listed are cited in Table 4,

The RGC‐RF counts obtained for our cohort from the OCT, Histology‐Balloon and PGRA methods are displayed alongside the results obtained for the same retinal eccentricity using the methods of Drasdo et al.,^22^ Watson^23^ and values from histology data^18^, ^19^, ^20^, ^21^ in Figure 6 and Table 4. The range of histology values obtained by Curcio and Allen for the retinal location of 2.32 mm retinal eccentricity used in this study are also included for reference in Figure 6 (taken from figure 8b^18^). The range of data at the specific test retinal eccentricity used in this study was not available for the other relevant published histology studies.

**TABLE 4:** RGC‐RF number underlying a Goldmann III stimulus calculated using methods assessed in this study (results displayed as median [IQR], grey cells), in comparison to previous histology estimates and values obtained from theoretical equations

	Nasal	Temporal	Superior	Inferior	Average across all meridians
Curcio^18^	153.0	157.8	112.4	94.2	129.4
Sjöstrand^19^	–	–	133.67		–
Dacey^20^	–	79.63	–	–	–
Tribble^21^	–	–	–	–	194.8
Drasdo (all)^22^	127.9	121.4	102.7	64.1	104.0
Drasdo^22^ (midget)	95.3	90.5	76.5	47.8	77.6
Watson^23^ (all)	123.5	120.6	73.2	88.0	101.7
Watson^23^ (midget)	92.1	89.0	54.6	65.6	75.9
Watson^23^ (ON‐midget)	46.0	45.0	27.3	32.8	37.8
PGRA	43.5 [30.9–50.6]	45.9 [33.3–55.6]	28.1 [24.0–38.3]	32.9 [28.0–40.0]	37.7 [33.8–46.0]
OCT‐model	129.7 [117.3–141.9]	107.7 [95.9–128.9]	92.2 [85.5–99.6]	84.0 [77.7–91.5]	105.3 [99.6–111.0]
Histology‐Balloon	133.2 [121.1–142.0]	121.6 [110.5–129.6]	90.6 [82.4–96.6]	84.7 [77.0–90.3]	107.5 [97.7–114.6]

*Note*: NB: Watson's ON‐midget subtype values are also representative of the OFF‐midget subtype too for the retinal location examined.

Abbreviations: IQR, inter quartile range; OCT, optical coherence tomography; PGRA, Peripheral grating resolution acuity.

The methods of Drasdo et al.^22^ and Watson^23^ provide estimates for all RGC subtypes, and the midget subtype only. It can be seen that values from histology^18^, ^19^, ^21^ and theoretical equations^22^, ^23^ counting all RGC subtypes are, on the whole, similar or higher than the median values from the OCT and Histology‐Balloon models. Midget‐only values from histology^20^ and theoretical equations^22^, ^23^ are lower than this but are still higher than the median PGRA values, which correspond most closely to the ON‐midget‐only estimates.^23^

## DISCUSSION

This study estimated RGC‐RF counts using three indirect methods and found that the count was highly dependent upon which method was used. The discrepancies in RGC‐RF count found between methods persisted even when the myopic individuals were taken out of the sample, suggesting that the findings were not just a result of many participants departing from the ‘normal’ structure of the emmetropic eye. The three main findings were as follows; (i) RGC‐RF counts obtained using the PGRA method were much lower (~60%) than both the OCT and Histology‐Balloon methods (Figure 4), (ii) intersubject variability in RGC‐RF count was evident for all methods (Figure 4) but was highest for the PGRA method and (iii) there was poor agreement between all three methods (Figure 5). These three points will now be discussed in more detail.

PGRA RGC‐RF counts were much lower than the findings from the other two methods, and several other histological estimates and the theoretical equations of Drasdo et al.^22^ and Watson^23^ (Figure 6). Our PGRA values are, however, similar to previous psychophysical data at the same or close test locations.^29^, ^53^ For example, when using Figure 2 in Wilkinson et al.^53^ to predict their findings at 8.1° eccentricity for each primary meridian, the average resolution acuity was ~8.75 c/deg, which is consistent with our average result of 8.58 c/deg. Matlach et al.^29^ reported an average RGC‐RF density at 8.8° of 3158 cells/mm^2^, which appears to agree well with our findings of a slightly higher RGC density, 3242 cells/mm^2^, at a slightly less peripheral test location for a younger, but more myopic, cohort.

The most likely explanation for the lower PGRA RGC‐RF counts compared with the other two methods is that high‐contrast PGRA measures likely recruit responses from only a subset of RGCs. By comparison, the OCT and Histology‐Balloon models are coarse measures incorporating all RGC subtypes and likely displaced amacrine cells in their counts. PGRA, on the other hand, will only count RGCs which are responding to the photopic, stationary, high spatial frequency grating stimulus. The previous literature suggests that midget RGCs predominantly respond to this stimulus, with good agreement being reported between PGRA measures and predicted visual acuity based on anatomical counts of midget RGCs.^20^, ^53^, ^55^ This hypothesis is also supported by Figure 6, which demonstrates a closer agreement between our PGRA values and the midget histology counts of Dacey,^20^ and midget‐specific estimates from Drasdo et al.^22^ and Watson,^23^ compared with counts including all RGC subtypes.^18^, ^19^, ^21^

While the agreement is improved, the PGRA‐derived RGC‐RF density estimates are still lower than the midget‐only histology estimates. It is probable that histological estimates of midget RGC‐RF count, and the equations based on them, are overestimations due to the difficulty in differentiating cell types histologically.^18^ Furthermore, some authors^56^, ^57^ advocate that PGRA is limited by the density of either the ON‐ or OFF‐midget RGCs (i.e., only approximately 50% of the midget RGC population). This 50% model could occur if: (a) either ON‐ or OFF‐midget RGCs only (not both) support resolution, (b) neighbouring ON and OFF RGCs function as a single sampling unit or (c) the ON and OFF RGCs sample the same retinal/stimulus locations, producing redundancy in the sampling arrays, with effectively only half the density of the total population.^53^ A 50% reduction in sampling density would lead to a √2 reduction in resolution acuity. Due to the anatomical arrangement of cone and midget RGCs across the retina, the 50% sampling model is most likely to apply to the fovea/parafoveal region (0–6 degrees) where each cone connects almost exclusively with one ON‐ and one OFF‐midget RGC.^58^ More peripherally, the two populations are more likely to sample at interspersed locations, leading to 100% of the midget RGC population determining PGRA.^53^ Experimentally, Wilkinson et al.^53^ found there was better correspondence between measured and predicted resolution acuity when the 100% model is used peripherally, but a 50% model is adopted for the foveal region. While the 50% hypothesis may not apply fully to our test location of 8.1°, there may be a continuum between 50% and 100%, with still less than 100% of midget RGCs responding at 8.1°. Wilkinson et al.^53^ also put forward the hypothesis that there was likely a continuum between the two extremes of 100% and 50% sampling. If a reduced percentage of midget RGC were responding to our PGRA stimulus, then this would help explain the lower RGC‐RF counts obtained from PGRA in this study. Indeed, when considering only the ‘ON subtype’ using the Watson^23^ density calculator, there was much better agreement with our PGRA values (Figure 6, Table 4).

While the RGC counts estimated using PGRA thresholds in this study show good agreement with Watson's ON‐midget RGC values^23^ (which are based on Curcio and Allen's mean histology data^18^), the assumption that such RGC counts are correct may be misplaced given the marked inter‐ and intra‐ study variability observed in the total RGC count for healthy observers from histology studies. Calculating the ratio of Curcio and Allen's mean total RGC count to the maximum reported within the study, and applying this ratio to the mean value of ON‐midget RGCs supplied by Watson, gives an expected maximum of 49.7 ON‐midget RGCs (mean 35.2). The same procedure for the minimum value gives an expected minimum of 23.4 ON‐midget RGCs. This range agrees well with our PGRA data, as illustrated in Figure 6.

We also considered how the mean ON‐midget RGC counts reported by Watson^23^ may vary given the disparity in total RGC mean count reported across different histology studies. For the vertical meridian, Sjöstrand et al.'s^19^ reported mean is 1.3‐times greater than that of Curcio and Allen.^18^ Applying the same ratio to Watson's ON‐RGC estimates, this would increase the mean values to 36.3 (Watson 27.9) and 42.7 (32.8) for the superior and inferior meridians, respectively. This corresponds to the 64th (superior) and 82nd (inferior) percentile of our PGRA values. Similarly, Trible et al. reported a mean total RGC count that is 1.5‐fold higher than Curcio and Allen's. Applying the same ratio to Watson's ON‐RGC estimates would increase the average peripheral value to 52.8 (35.2), which corresponds to ~91st percentile of our PGRA values. The disparity between histology accounts is likely to be a consequence of different aged donors, methodological challenges associated with obtaining accurate counts and natural intersubject variability.

Indeed, what is clear from both published histology data and our experimental data for all three methods is that RGC‐RF count can vary significantly from individual to individual. This demonstrates the unsuitability of using an average count (e.g., from the theoretical models of Drasdo et al.^22^ and Watson^23^) if interested in knowing the RGC‐RF count for an individual (e.g., to examine structure–function relationships). Average peripheral RGC‐RF counts varied in our cohort by a range of about 1.5‐fold for both the OCT‐model and Histology‐Balloon model and by approximately 15‐fold for the PGRA measures. Given that intersubject variability in RGC number is known to vary with retinal location,^18^ we attempted to compare these values to estimates from histology data at, or as close as possible, to the study's value of retinal eccentricity (2.32 mm). Examining figure 8b from Curcio and Allen,^18^ we estimated the intersubject range at our study location as 2.1‐fold, with the maximum and minimum values illustrated in Figure 6. For Sjöstrand et al.'s data,^19^the intersubject range was 1.5‐fold at 2.4 mm eccentricity (Table 1^59^), and for Tribble et al.'s data,^21^ we estimated a 3.1‐fold range for healthy observers at ~2.9 mm eccentricity (Tribble et al. figure 1). Individual RGC density values were not included by Dacey.^20^

At first glance, the intersubject variability of RGC‐RF estimates for the OCT‐model and Histology‐balloon model (both ~1.5 fold) appear to be more consistent with previous histology reports (range 1.5‐fold to 3.1‐fold across studies) compared with estimates generated using PGRA results (~15‐fold). This similarity is perhaps unsurprising given that histology data forms the basis of both models.

Functional PGRA measurements may be more variable and prone to human error given the subjective nature of the task, requiring participant understanding, cooperation and attention. Indeed, our findings agree with previous work reporting greater variability for RGC estimates obtained from functional visual field sensitivity data, compared with OCT‐derived estimates.^14^ While a two‐alternative forced choice paradigm was used to minimise the effect of observer criterion when measuring PGRA thresholds, should lapses in attention/cooperation occur during the subjective PGRA measures, then this could result in more variable RGC counts compared with the purely objective structural measures involved in the OCT‐model and Histology‐balloon model counts.

However, it is not possible to conclude that the range of PGRA values is inaccurate in the absence of a ground‐truth measurement of RGC‐RF in our study population. As such, it may be that the range in RGC‐RF counts found for PGRA in the present study are more reflective of the ground truth in our cohort rather than being artefactual owing to measurement variability. Given the larger cohort (*n* = 44, compared with *n* = 3–6 in studies reporting histological RGC counts), coupled with the wide range of refractive errors in the current study, it would be reasonable to expect that intersubject variability might be higher than previous histological reports. Indeed, when considering the intersubject variability for myopic and non‐myopic participants separately, there was a greater spread in the myopic participants for all three methods. This would perhaps be expected given the wider ranges of refractive error (9.25 D vs. 2.00 D in the myopic and non‐myopic groups, respectively) and axial length range (5.46 vs. 3.15 mm in the myopic and non‐myopic groups, respectively). The largest difference in intersubject variability was observed for PGRA, where there was about a 13‐fold range in myopes and around a 4‐fold range in non‐myopes. Large degrees of intersubject variability were also found for myopic participants in Chui et al.^1^ Converting their resolution acuity results (c/deg) into the RGC number underlying GIII using the same methodology as used for the PGRA measures here, a 13.9‐fold and 7.6‐fold intersubject variability existed for their myopic participants (refractive range −0.50 D to −14.25 D) at 10 degrees eccentricity in the nasal and temporal retina, respectively. This high intersubject variability for the RGC count obtained using PGRA with myopic participants could point towards myopia influencing *functional* midget RGC density (measured with PGRA) over and above what is expected from retinal stretch and axial length growth (considered in both the OCT and Histology‐balloon models). It has previously been suggested that retinal dysfunction may occur in myopia, perhaps as a result of damage to the visual neurons during the retinal stretch.^1^, ^57^, ^60^ This may explain the wider range of results obtained for the myopic group using PGRA. Whereas the OCT‐ and Histology‐balloon models would only be affected by structural differences (ocular growth and retinal stretch) within the myopic eyes, the PGRA measures could be affected by *both* structural and functional differences between participants.

As well as greater intersubject variability, PGRA values also show more intraretinal variability (i.e., greater differences in RGC‐RF across the four meridians) than the other two models. On average, functional PGRA measures varied over a 1.9‐fold range across the retina, compared with an average 1.6‐fold difference for both structure‐based models. In addition, the degree of intraretinal variability also showed the most intersubject variability for PGRA. This agrees with the previous literature that suggests substantial between‐subject variability in the shape of the visual field,^61^ this being greater than the observed variations in the RGC number. Such discrepancies between how functional and structural measures vary across the visual field may also help to explain the poor agreement between functional (PGRA) and structural (OCT‐model, Histology‐balloon) methods used in this study. Previous work has shown that for conventional perimetric contrast thresholds, the change in spatial scale in the nasal visual field is more shallow than the decline in ganglion cell number,^62^ so that agreement between functional measures and RGC number is also likely to vary across locations within the visual field.

Given such discrepancies between functional and structural measures, and considering that PGRA is likely to target a different population of cells than the other two methods, it is perhaps unsurprising that poor agreement was observed between functional PGRA counts and those from the structural models. However, an imperfect agreement was also found between the OCT‐ and Histology‐Balloon models (Figure 5), despite both including normative histological measures of RGC density as the basis for their calculations and a low mean difference. Thus, for a given individual, RGC‐RF count could be near the top of the range for one measure and near the bottom for the other. Both models share a common methodology when creating the personalised histology map. However, the OCT‐model also utilises a co‐localised measurement of structure (RGCL thickness at the test location), rather than just assuming retinal stretch occurs uniformly throughout the globe (global expansion), as is the case for the Histology‐Balloon model. One would expect that if the global expansion model is appropriate, then the agreement between the OCT and Histology‐Balloon models should be good. However, as this was not the case, with wide LOA observed with Bland–Altman analysis (Figure 5), this may suggest that the global expansion model is not appropriate for all of our study population. Indeed, previous structural^63^, ^64^, ^65^ and functional^1^, ^2^, ^66^, ^67^ data provide evidence *against* a global expansion model. Other models of myopic expansion include equatorial expansion, whereby growth is localised to the equatorial region of the globe and posterior pole expansion, in which growth is localised to that region.^66^ Atchison et al.^68^ found that no single expansion model could define their entire myopic population, meaning that any ‘one‐model‐fits‐all’ approach is too simplistic. However, in the absence of a measure of peripheral ocular shape in the current cohort, we are unable to test the hypothesis that departures from a global expansion result in poor agreement between the OCT and Histology‐Balloon models.

**FIGURE 7:**
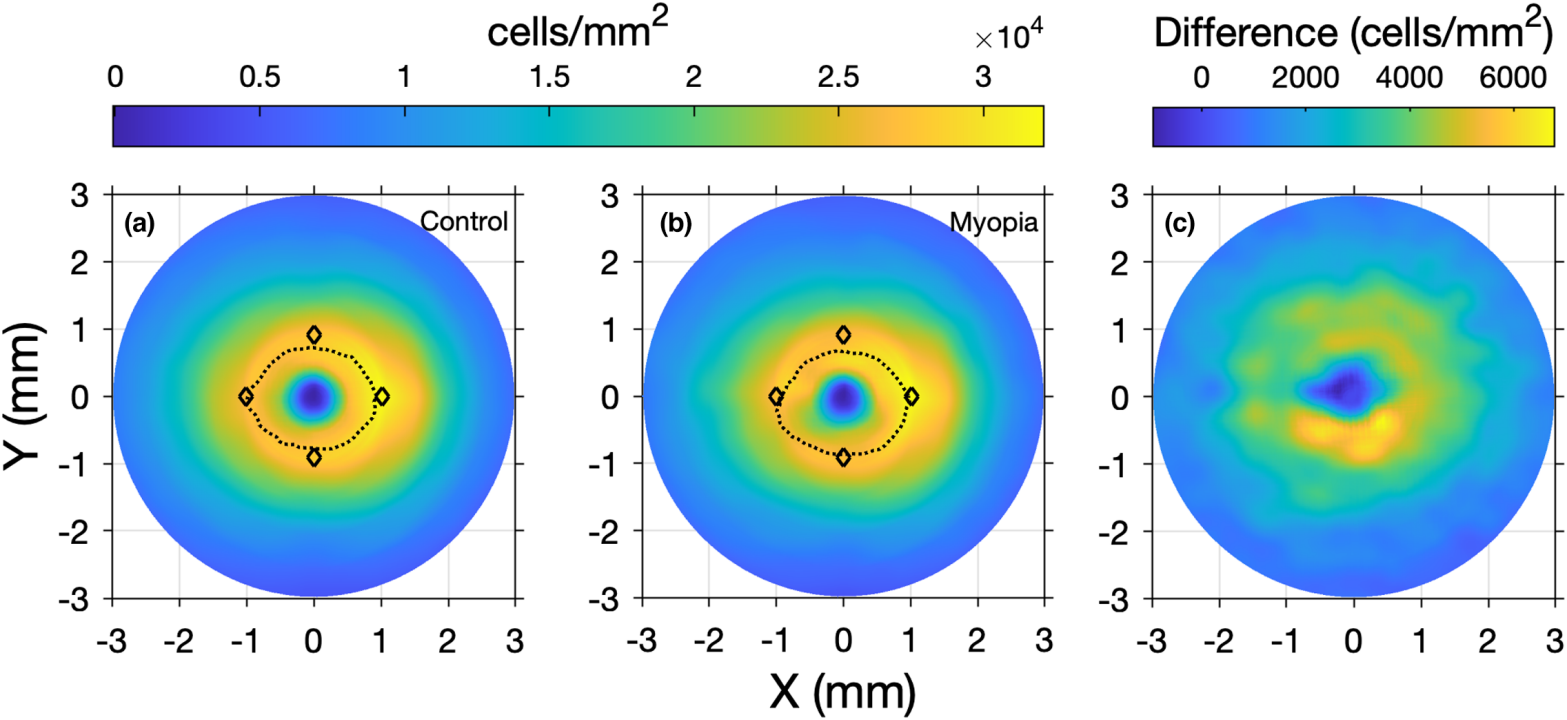
Mean retinal ganglion cell receptive field (RGC‐RF) density measures generated using optical coherence tomography (OCT) measures of retinal ganglion cell layer (RGCL) thickness in the control (a) and myopic (b) observers. Peak density is included as a dashed line in each plot, with the mean peaks reported for the histology data of Curcio and Allen (diamonds)^18^ included for reference. The difference in OCT‐derived density values between myopes and nonmyopes is shown in Figure 7c.

Another potential explanation for the poor agreement between these models is simply that, for any given individual, one or both estimates of RGC‐RF may be inaccurate as a result of the limitations and multiple assumptions associated with each method. Firstly, both methods rely on interpolated data from a small sample histology study,^18^ which creates issues from the outset given large intersubject variability in RGC number. While attempts were made to make the histology map more ‘personalised’, by stretching according to a global expansion model (also used by Montesano et al.^46^), compared with the original method of Raza and Hood,^14^ this may not be a suitable expansion model for all individuals, and there may be additional anatomical differences between an individual eye and the histology data that are not accounted for. For example, the spatial localisation and shape of the RGC‐layer profile may differ when compared with the histology data. This is illustrated in Figure 7, which demonstrates the differences in average RGC peak shape of the histology data^18^ from that for our cohort, derived using actual OCT data, for both control (Figure 7a) and myopic (Figure 7b) participants. In addition, differences may be exacerbated by the axial length of a given eye, with RGC profile changing as a result of retinal stretch. Indeed, Figure 7a,b show that the peak shape is different for controls and myopes. Figure 7c demonstrates that the difference in RGC density between myopes and controls is nonuniform around the fovea, providing further indirect evidence for a difference in RGC density and peak location between the two refractive groups. The work of Montesano et al.^46^ also suggests differences in RGC peak profile between myopes and controls. As such, using the same, average histology map for all participants with varying axial lengths could lead to inaccuracies.

Another assumption made by the OCT‐model is that the RGC layer is composed entirely of RGCs, with Raza and Hood^14^ themselves suggesting that this is an oversimplification and thus a limitation of their methodology. Specifically, OCT measures of RGC‐layer thickness can include displaced AC and other non‐neural components (e.g., glial tissue, vasculature). As discussed previously, this may also help to explain the findings of the current study whereby a larger RGC‐RF count was obtained using the OCT compared with the PGRA method, in which only functional RGCs respond to the stimulus. Similarly, histological numbers may also include ACs within the ‘RGC’ count, with Curcio and Allen^18^ highlighting the challenge in distinguishing RGCs from displaced ACs, particularly within the central 3 mm. The authors suggest that this issue could have led to an underestimation of ACs by up to 47% and an overestimation of RGCs by nearly 14%, which would apply equally to any study or model using their data.^14^, ^16^, ^22^, ^23^, ^34^, ^35^

There are also assumptions in both models regarding the interpolation of the histological data and calculating RGC displacement. The magnitude of RGC cell body displacement from underlying photoreceptors (Henle fibre length) varies across histological studies,^19^, ^22^, ^23^ likely due to small sample sizes and individual variance. The displacement model proposed by Montesano et al.^46^ was used in this study for both the OCT and Histology‐Balloon models; calculating displacement on an individualised basis and accounting for the scaling of retinal structures with axial length. Regardless, inherent assumptions remain, including assuming a global model of expansion with axial length. Only PGRA, which directly measures the functional response of the RGCs, can negate the assumptions regarding RGC displacement completely. It may be that potential error in both the OCT‐ and Histology‐Balloon models accumulates with each assumption drawn into the mix, resulting in inaccurate estimations of RGC‐RF count and poor agreement when considering the counts on an individual basis. However, as mentioned previously, without the ability to measure the RGC‐RF count directly (which is the very reason these alternative, ‘surrogate’ measures exist in the first place), it is not possible to ascertain the accuracy of the values.

In summary, this study demonstrates poor agreement between all three indirect measures of RGC‐RF count. Consequently, it is not possible for researchers to compare counts from previous literature that utilise different methods. Given that RGC‐RF counts are ‘non‐transferable’ between methods, it is vital that the same method is utilised at all time points when trying to monitor the RGC‐RF count over time. Future work should look at the precision of the various methods used to obtain the RGC‐RF count, to evaluate which method may be most appropriate for both comparing differences between target groups and longitudinal studies investigating changes in the RGC‐RF count over time.

## CONCLUSIONS

This study demonstrated that indirect estimates of RGC‐RF count are highly dependent upon the method used to obtain them. PGRA estimates were markedly lower than those obtained using the OCT‐ or a Histology‐Balloon model, likely due to only a subset of RGCs responding to the PGRA grating stimulus (as opposed to the other two models that included all RGC subtypes, and non‐neural elements such as AC). Intersubject variability was evident using all techniques and was largest for the PGRA method. In the absence of a direct ‘reference‐standard’ method of obtaining RGC‐RF counts in the cohort (the very reason these alternative methods exist), it is not possible to ascertain which method produces the most accurate count, with each having limitations. Based on our results, however, we now know that there is poor agreement between these techniques, and therefore, RGC‐RF counts should not be compared between studies utilising different methods. Further, any researcher wanting to monitor RGC‐RF count longitudinally must use the same method throughout. Future work should investigate the precision of these methods to ascertain which would be best to use in longitudinal work.

## AUTHOR CONTRIBUTIONS

**Victoria Stapley:** Conceptualization (equal); formal analysis (lead); investigation (lead); methodology (equal); project administration (lead); visualization (lead); writing – original draft (lead); writing – review and editing (equal). **Roger S Anderson:** Conceptualization (equal); methodology (equal); supervision (equal); writing – original draft (supporting); writing – review and editing (equal). **Kathryn Saunders:** Supervision (equal); writing – original draft (supporting); writing – review and editing (equal). **Padraig J Mulholland:** Conceptualization (lead); methodology (equal); software (lead); supervision (lead); visualization (equal); writing – original draft (supporting); writing – review and editing (equal).

## CONFLICT OF INTEREST

The authors report no conflicts of interest and have no proprietary interest in any of the materials mentioned in this article.
